# Genetic similarities between *Cyclospora cayetanensis* and cecum-infecting avian *Eimeria* spp. in apicoplast and mitochondrial genomes

**DOI:** 10.1186/s13071-015-0966-3

**Published:** 2015-07-08

**Authors:** Kevin Tang, Yaqiong Guo, Longxian Zhang, Lori A. Rowe, Dawn M. Roellig, Michael A. Frace, Na Li, Shiyou Liu, Yaoyu Feng, Lihua Xiao

**Affiliations:** Division of Scientific Resources, Centers for Disease Control and Prevention, Atlanta, GA 30333 USA; State Key Laboratory of Bioreactor Engineering, School of Resources and Environmental Engineering, East China University of Science and Technology, Shanghai, 200237 China; Division of Foodborne, Waterborne, and Environmental Diseases, Centers for Disease Control and Prevention, Atlanta, GA 30333 USA; College of Animal Science and Veterinary Medicine, Henan Agricultural University, Zhengzhou, 450002 China

**Keywords:** *Cyclospora*, Genomics, Genome, Apicoplast, Mitochondria, Molecular diagnosis

## Abstract

**Background:**

*Cyclospora cayetanensis* is an important cause for diarrhea in children in developing countries and foodborne outbreaks of cyclosporiasis in industrialized nations. To improve understanding of the basic biology of *Cyclospora* spp. and development of molecular diagnostic tools and therapeutics, we sequenced the complete apicoplast and mitochondrial genomes of *C. cayetanensis*.

**Methods:**

The genome of one Chinese *C. cayetanensis* isolate was sequenced using Roche 454 and Illumina technologies. The assembled genomes of the apicoplast and mitochondrion were retrieved, annotated, and compared with reference genomes for other apicomplexans to infer genome organizations and phylogenetic relationships. Sequence variations in the mitochondrial genome were identified by comparison of two *C. cayetanensis* nucleotide sequences from this study and a recent publication.

**Results:**

The apicoplast and mitochondrial genomes of *C. cayetanensis* are 34,155 and 6,229 bp in size and code for 65 and 5 genes, respectively. Comparative genomic analysis showed high similarities between *C. cayetanensis* and *Eimeria tenella* in both genomes; they have 85.6 % and 90.4 % nucleotide sequence similarities, respectively, and complete synteny in gene organization. Phylogenetic analysis of the genomic sequences confirmed the genetic similarities between cecum-infecting avian *Eimeria* spp. and *C. cayetanensis*. Like in other coccidia, both genomes of *C. cayetanensis* are transcribed bi-directionally. The apicoplast genome is circular, codes for the complete machinery for protein biosynthesis, and contains two inverted repeats that differ slightly in LSU rRNA gene sequences. In contrast, the mitochondrial genome has a linear concatemer or circular mapping topology. Eight single-nucleotide and one 7-bp multiple-nucleotide variants were detected between the mitochondrial genomes of *C. cayetanensis* from this and recent studies.

**Conclusions:**

The apicoplast and mitochondrial genomes of *C. cayetanensis* are highly similar to those of cecum-infecting avian *Eimeria* spp. in both genome organization and sequences. The availability of sequence data beyond rRNA and heat shock protein genes could facilitate studies of *C. cayetanensis* biology and development of genotyping tools for investigations of cyclosporiasis outbreaks.

## Background

Human cyclosporiasis is caused by *Cyclospora cayetanensis*. It is endemic in many developing countries, responsible for significant morbidity in children and AIDS patients [1, 2]. It is also a major cause of foodborne diarrheal illnesses in industrialized nations, especially in North America. Since the mid-1990s, numerous outbreaks of cyclosporiasis have occurred in the United States and Canada, mostly associated with fresh produce imported from Mexico and Central America [2, 3]. The lack of molecular diagnostic tools for case-linkage and trace-back investigations of outbreaks has hampered responses from public health and regulatory agencies such as the U.S. Food and Drug Administration and Centers for Disease Control and Prevention. This was exemplified in two recent large multistate outbreaks of cyclosporiasis in 2013 and 2014 in the United States [4] (http://www.cdc.gov/parasites/cyclosporiasis/outbreaks/index.html).

One major reason for the lack of molecular diagnostic tools and poor understanding of the basic biology of *C. cayetanensis* is the limited availability of nucleotide sequences from the parasite. At the end of 2014, among the ~500 entries of *Cyclospora* sequences in GenBank, almost all of them were from the rRNA (SSU rRNA, 5.8S rRNA, ITS-1, and ITS-2) and 70 kDa heat shock protein (HSP70) genes. The conserved sequence nature of rRNA and HSP70 genes and intra-isolate variations among different copies of ITS-1 and ITS-2 make the development of genotyping tools for the parasite difficult [5–9]. There is a need for genomic sequencing to identify polymorphic markers and promote biologic studies of *C. cayetanensis* [10].

Apicoplasts and mitochondria are two important intracellular organelles of apicomplexan parasites [11]. The apicoplast is the result of secondary endosymbiosis by ancient apicomplexan parasites, and harbors metabolic pathways that are necessary for the survival of parasites but are very distinct from those of host species [12]. Likewise, mitochondria play key roles in energy metabolism in apicomplexan parasites [11]. Most apicomplexan parasites, with the notable exception of *Cryptosporidium* spp., have both apicoplast and mitochondrial genomes. Because of the prokaryotic origins and biological importance of both apicoplasts and mitochondria, they have been widely used as drug targets against apicomplexan parasites [12–15]. The non-recombining and co-inherited nature of apicoplast and mitochondrial genomes has recently been used in the development of barcoding tools for tracking worldwide migration of *Plasmodium* spp. [16–18]. Even at the species level, a recent barcoding study of *Eimeria* spp. has shown that partial mitochondrial cytochrome c oxidase subunit I (cox1) sequences are more reliable species-specific markers than complete nucleic SSU rRNA sequences, as they provide more synapomorphic characters [19]. The near complete genome of *C. cayetanensis* has been published recently [20].

As there are no data on the apicoplast genome and only partial data on the mitochondrial genomes of *C. cayetanensis*, we have characterized in this study the complete apicoplast and mitochondrial genomes of an isolate from China. Data generated have shown a close relatedness in apicoplast and mitochondrial genomic sequences between *C. cayetanensis* and cecum-infecting avian *Eimeria* spp. and nucleotide sequence polymorphism in mitochondrial genomes between the isolate in this study and the one in the recent publication. These sequence data should be useful in improving our understanding of the biology of *C. cayetanensis* and developing advanced molecular detection tools.

## Methods

### DNA preparation, sequencing and de novo assembly

DNA was extracted from a *C. cayetanensis* specimen collected in July 2011 from a patient in Kaifeng, Henan, China. The identification of *C. cayetanensis* was done by acid-fast microscopy and confirmed by PCR and sequence analyses of the SSU rRNA gene as previously described [5]. DNA was extracted from sucrose and cesium chloride gradient-purified oocysts using the QIAamp®DNA Mini kit (Qiagen, Valencia, CA). The genome of *C. cayetanensis* was sequenced using the Roche 454 GS-FLX Titanium (Roche, Branford, CT), with average read lengths of 400–450 bp. Supplemental 100 × 100 bp paired-end sequencing was completed using the Illumina Genome Analyzer IIx (Illumina, San Diego, CA). After sequencing, 454 sequence reads were assembled using Roche Newbler (gsAssembler 2.3). Illumina reads were trimmed using clc_quality_trim within the CLC Assembly Cell v 4.1.0 (http://www.clcbio.com/) with minimum quality score of 20 and minimum read length of 70. Trimmed reads were assembled de novo with the clc_assembler of the CLC Assembly Cell. Contigs for *C. cayetanensis* apicoplast and mitochondrion genes were identified by blasting the assemblies against the GenBank database.

### PCR confirmation

PCR amplification and sequencing of the amplicons were used to confirm the inverted repeats in the plastid genome. Two pairs of primers were used to amplify the regions that join the inverted repeat to the main body of the apicoplast. The primer sets were F1: 5′-AGT CGC TAA GTA GCC AAG TTT-3′ and R1: 5′-TTG TCT TGC CTG TGC TAT AGT AAT-3′, and F2: 5′- ACT ACA TCA ACG GCT AAC T-3′ and R2: 5′-GTA CGA GAG GAC CAA AGA AA-3′, which targeted a 634 (between rps4 and LSU rRNA genes) and 743 bp fragment (between ycf24 and LSU rRNA genes), respectively. The reactions contained 1 μl of DNA, 1× GeneAmp PCR buffer (Applied Biosystems, Foster City, CA), 1.5U of GoTaq polymerase (Promega, Madison, WI), 250 μM dNTPs (Promega), 3 mM MgCl_2_, 250 nM of each primer, and 400 ng/μl of non-acetylated bovine serum albumin (Sigma-Aldrich, St. Louis, MO). The amplification was performed on a GeneAmp PCR 9700 thermocycler (Applied Biosystems), consisting of an initial denaturation at 94 °C for 5 min; 35 cycles at 94 °C for 45 s, 55 °C for 45 s, and 72 °C for 1 min; and a final extension at 72 °C for 7 min. The secondary PCR products were detected by 1.5 % agarose gel electrophoresis, and sequenced in both directions using the BigDye Terminator V3.1 Cycle Sequencing Kit (Applied Biosystems) on an ABI 3130xl Genetic Analyzer (Applied Biosystems).

### Genome annotation and comparison

As the apicoplast and mitochondrial genomes of *C. cayetanensis* were in complete synteny with those of *Eimeria* spp., we used the Rapid Annotation Transfer Tool (RATT) [21] to transfer the annotations from the *E. tenella* apicoplast genome (AY217738) and *E. dispersa* mitochondrion genome (KJ608416). Potential genes and open reading frames (ORFs) were also identified using GeneMark [22]. The annotation was then manually improved by comparing the predicted ORFs and RATT annotations.

### Detection of sequence variations

The 454 and Illumina sequence reads were mapped to reference genomes and assembled sequence contigs using the CLC Genomics Workbench 7.0.3 (http://www.clcbio.com/). The probabilistic variant detection tool in the software package was used to detect single nucleotide variations (SNVs) among mapped reads, using the parameters of min coverage 10, probability 90, required variant count 2, and all other filters unchecked. In-house perl scripts were developed to generate SNV data for display. The assembled apicoplast and mitochondrial genomes were further compared with the corresponding reference genomes using Nucmer within the MUMmer package [23].

### Phylogenetic analysis

Full genome sequences for apicoplasts and mitochondria of other apicomplexans were extracted from GenBank and aligned with *C. cayetanensis* sequences by using the sequence alignment function within the CLC Genomics Workbench 7 (http://www.clcbio.com/). Poorly aligned positions in the alignments were eliminated by using Gblocks [24]. The maximum likelihood phylogenies of these sequences were inferred from the data using the CLC Genomics Workbench under the general time reversible model of nucleotide substitution. The confidence of the cluster formation was assessed by using bootstrap analysis with 1,000 replicates.

### Nucleotide sequence accession numbers

Nucleotide sequence data from the whole genome sequencing, including the SRA data and assembled contigs, were submitted to NCBI BioProject PRJNA256967. Sequences of the annotated apicoplast and mitochondrial genomes were deposited in the GenBank database under accession numbers KP866208 and KP796149.

## Results

### Genome coverage and copy numbers

Altogether, 46.8 Mb of nucleotides in 4,811 assembled contigs (N50 = 55,741 bp) were obtained from the whole genome sequencing by 454 and Illumina technologies. Blast analysis of the sequences identified sequences of the complete apicoplast and mitochondrial genomes of *C. cayetanensis*. Results of contig assembly and read mapping indicated the ratio of the copy numbers of nucleic and apicoplast genomes was 1.00:1.08 (average coverage = 205.03 and 220.62 folds for nucleic and apicoplast genomes, respectively). In contrast, the copy number ratio for nucleic and mitochondrial genomes was 1.00:513.25 (average coverage = 205.03 and 10,5231.79 folds for nucleic and mitochondrial genomes, respectively).

### Apicoplast genome

#### Genome organization

**Fig. 1:**
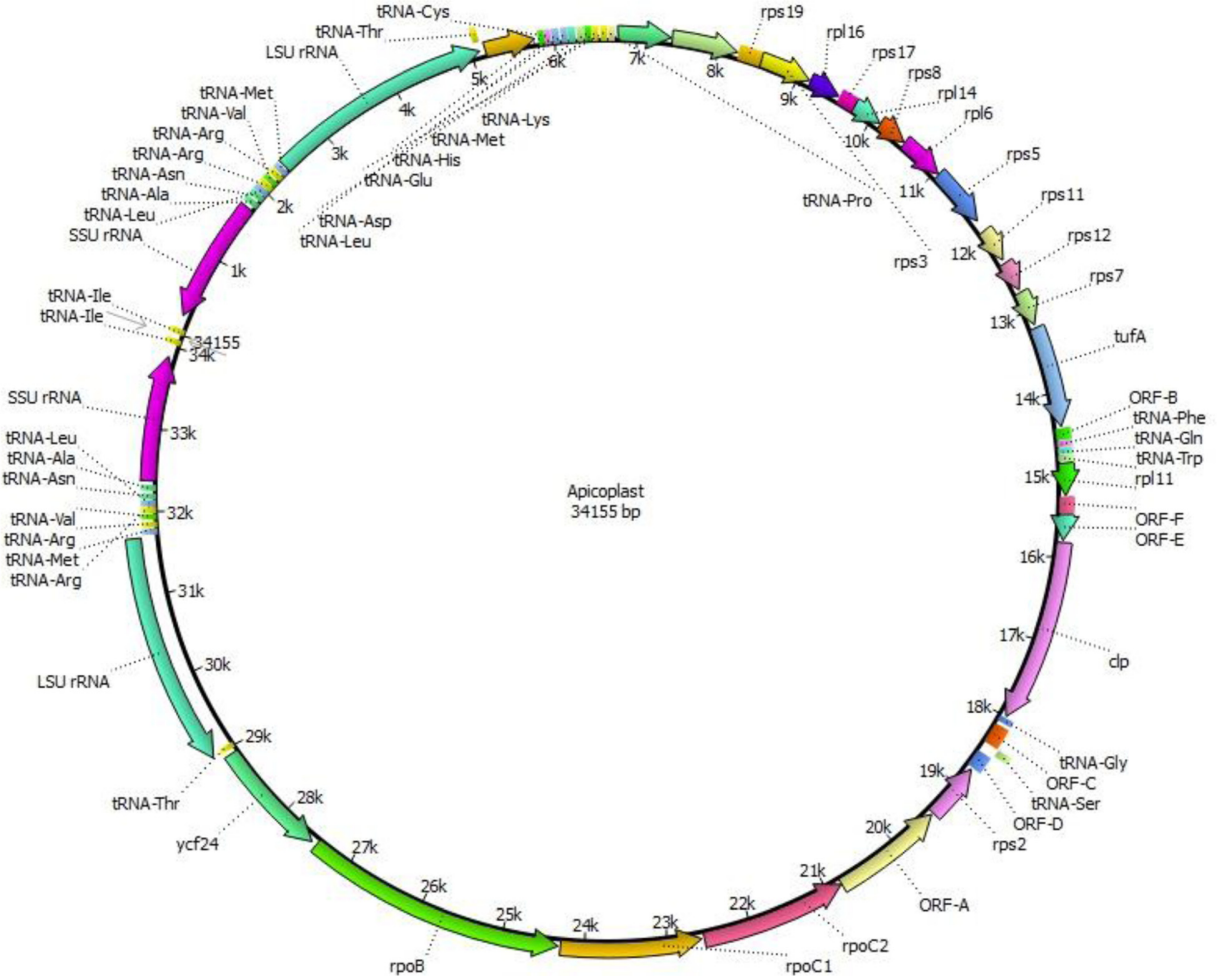
Map of the apicoplast genome of *Cyclospora cayetanensis*

**Fig. 2:**
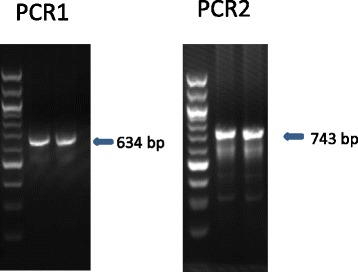
PCR analysis of the regions joining the inverted repeat (IR) and the remainder of the *C. cayetanensis* apicoplast. A 634 bp and a 743 bp fragment covering the joints of the IR and the remainder of the apicoplast were amplified by using PCR with the one primer in the repeat and the other primer in the other part of the apicoplast. The molecular weight marker is a100bp DNA ladder

The apicoplast genome is 34,155 bp in size with the following base composition: A (40.28 %), T (37.76 %), C (10.79 %) and G (11.16 %), with an overall AT content of 78.04 %. It contained two inverted repeats (IR). Each IR unit is 5,244 bp in length and contains genes coding for an SSU rRNA, an LSU rRNA, and nine tRNAs (Fig. 1). PCR with one primer in the repeat and the other primer in the non-repeat part of the apicoplast sequence amplified the two regions joining the IR to the main body of the apicoplast (Fig. 2). The joint sequences were confirmed by Sanger sequencing, which yielded sequences identical to those from the whole genome sequencing. PCR amplification of the sequence between the closer ends of the two IRs was not successful probably because of the inverted nature of the repeat units. However, a search of reads from Roche 454 sequencing revealed that there are 33 bp between the closer ends of the two IRs. This allowed the construction of the full circular apicoplast genome of *C. cayetanensis*. The presence of two IRs was also confirmed by mapping of sequence reads to the assembled contigs, as the two IRs differed by eight nucleotides in a 58-bp region of the LSU rRNA gene (Table 1). The relative placement of the two slightly different IR units in the circular apicoplast genome was not clear.

**Table 1:** Sequence differences between two inverted repeats (IR) of the apicoplast genome of *Cyclospora cayetanensis* ^a^

Reference position	Reference	Allele^b^	Count	Coverage (fold)	Allele frequency (%)	Mean read quality score
4096	C	-	272	413	65.86	28.94
4098	A	C	265	414	64.01	30.84
4100	A	G	271	420	64.52	30.46
4117	G	A	300	462	64.94	30.19
4136	G	A	276	417	66.19	31.3
4142	A	T	241	380	63.42	27.8
4149	C	T	210	340	61.76	30.39
4153	A	T	198	324	61.11	29.71
29970	T	A	171	302	56.62	30.46
29974	G	A	180	320	56.25	31.23
29981	T	A	218	358	60.89	28.16
29987	C	T	249	390	63.85	30.38
30006	C	T	275	418	65.79	30.59
30023	T	C	241	388	62.11	30.16
30025	T	G	240	387	62.02	30.48
30027	G	-	246	385	63.90	28.14

^a^The variable fragment is located in a 58-bp region (5′- CTATAACGGTCCAAAGGTAGCGAAATTCCTTGTCGGGTAAGTTCCGACCTGCACGAA-3′) of the LSU rRNA gene of each IR

^b^Dash indicates a nucleotide deletion

#### Gene organization

The annotation of the sequences indicated that there are 65 genes coded by the apicoplast genome, including 4 rRNA genes, 28 protein-coding genes, and 33 tRNA genes for all 20 amino acids. The protein-coding genes include 6 genes for ribosomal protein large subunit (rpl), 10 genes for ribosomal protein small subunit (rps), 3 genes for RNA polymerase (rpo), 6 genes for hypothetical proteins, and 1 gene each for elongation factor Tu (tufA), ATP-dependent Clp protease (clpC) and putative ABC transporter (ycf24). The *C. cayetanensis* apicoplast has almost all the genes encoded in the *E. tenella* apicoplast genome except rpl36, which is truncated. Similarly, the orf-D gene of *C. cayetanensis* codes for only 70 amino acids in length, which is 29 amino acids shorter than that of *E. tenella*. orf-A codes for a protein similar to the DNA-directed RNA polymerase subunit. The organization of these genes is shown in Fig. 1.

#### Usage of start and stop codons

Rpl2, rps11 start with the codon ATT and rpl11 starts with codon ATA. The start codon for rpoC1 is unclear. By comparing the same gene in *E. tenella*, the corresponding start codon of rpoC1 in *Cyclospora* is TTT. The start codon for all remaining genes is ATG. A total of 20 in-frame TGAs coding for tryptophan (W) are present in 14 genes in the apicoplast genome. Because of the unavailability of TGA as a stop codon, most apicoplast genes in *C. cayetanensis* end with the codon TAA, with three genes (rpl2, orf-C, and rpoB) using TAG (data not shown).

#### Genetic similarity to other apicomplexans

**Fig. 3:**
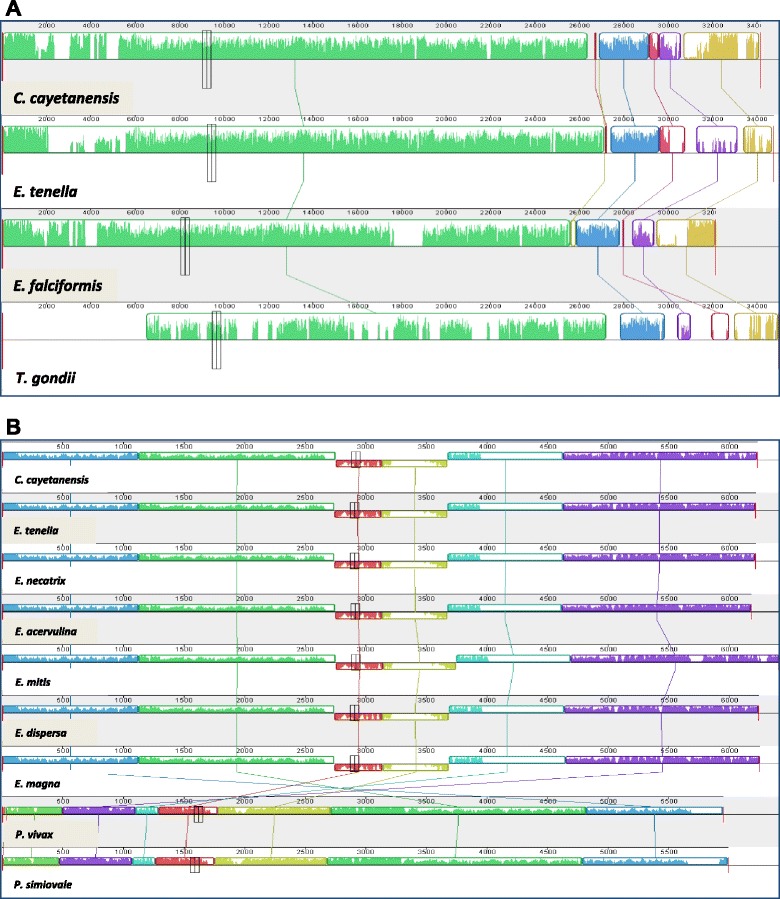
Synteny in gene organizations between *Cyclospora cayetanensis* and *Eimeria* spp. in the apicoplast (**a**) and mitochondrial (**b**) genomes. The color blocks are conserved segments of sequences internally free from genome rearrangements, whereas the inverted white peaks within each block are sequence divergence between the *C. cayetanensis* genome and other genomes under analysis

**Fig. 4:**
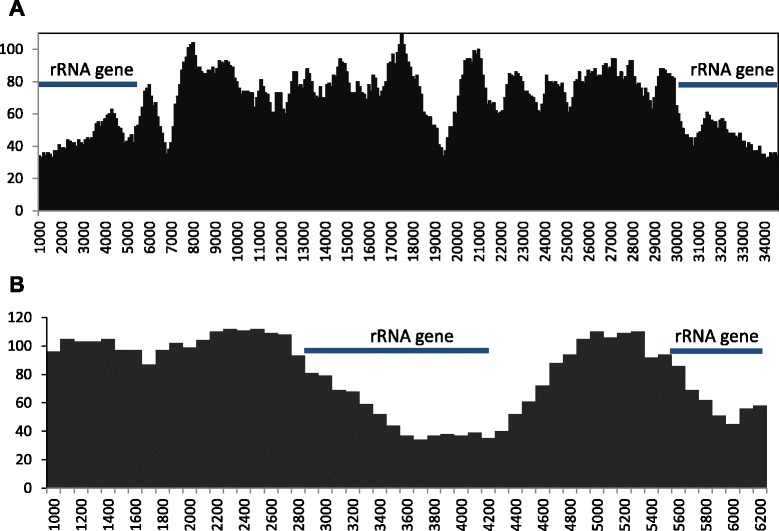
Distribution of SNVs along apicoplast (**a**) and mitochondrial (**b**) genomes in comparison with those of *Eimeria tenella*. The number of SNVs was calculated by mapping of sequence reads to the reference *E. tenella* genome in a sliding window of 1,000 bp with 100-bp steps

A Blast search of the NCBI database with the *C. cayetanensis* apicoplast sequence showed that the sequence was most similar to the apicoplast genome of *E. tenella* (access no. AY217738.1). Genome sequence alignments showed that the *C. cayetanensis* apicoplast genome has complete gene synteny to the apicoplast genome of *E. tenella* and other *Eimeria* spp. and good synteny to the genome of *T. gondii* (Fig. 3a). Aligning the assembled apicoplast genome sequence to that of the *E. tenella* apicoplast genome with NUCmer resulted in a single alignment that covers 99.85 % of the apicoplast genome, confirming the synteny between the two genomes. The identity between the two genomes in the aligned regions was 85.6 %. The sequence divergence between the two genomes was mostly 8–10 % as calculated in a sliding window of 1,000 bp in sequence read mapping. However, much lower sequence differences were seen in rRNA genes within the two IR units (Fig. 4a). SNV analysis by read mapping did not identify any intra-isolate sequence polymorphism beyond what was described between the two IRs.

**Fig. 5:**
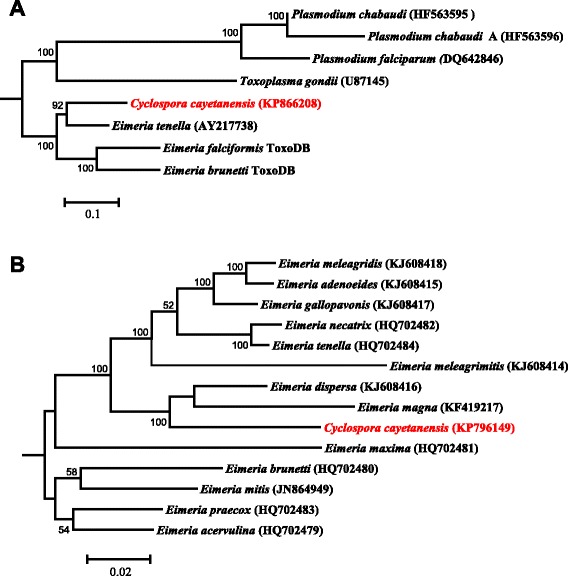
Maximum likelihood phylogeny of apicoplast (**a**) and mitochondrial (**b**) genomes. The phylogeny was inferred under the general time reversible model of nucleotide substitution in the CLC Genomics Workbench with the Gblocks-processed aligned regions. The numbers at nodes indicate bootstrap values from 1,000 replicates. The scale bars indicate the estimated substitutions per site

In a maximum likelihood analysis of apicoplast genome sequences from apicomplexans, *C. cayetanensis, E. tenella*, *E. falciformis*, and *E. brunetti* formed one clade that was divergent from *T. gondii* and *Plasmodium* spp. Within the clade formed by *Cyclospora* and *Eimeria*, *C. cayetanensis* clustered together with *E. tenella*, confirming the genetic similarity between the two species based on direct sequence comparison and gene annotations (Fig. 5a).

### Mitochondrial genome

#### Genome organization and gene content

**Fig. 6:**
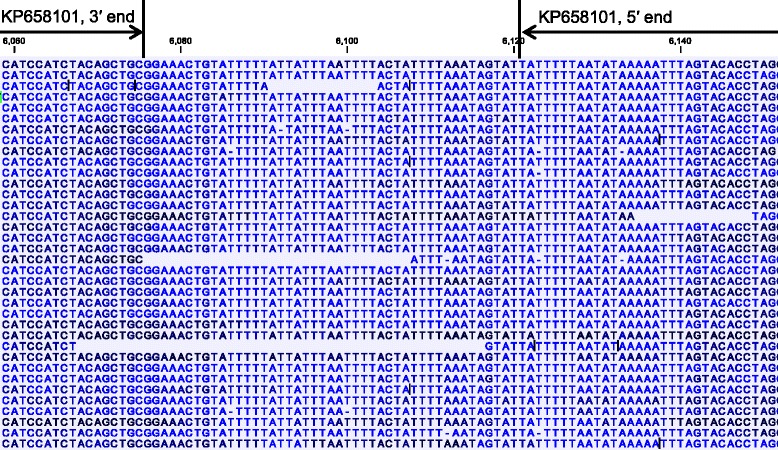
Nucleotide sequences at the joint region of the concatenated mitochondrial genomes of *Cyclospora cayetanensis* as shown by the result of sequence read mapping. Roche 454 sequence reads were mapped to the assembled *C. cayetanensis* mitochondrial genome from CHN_HEN01 (KP796149). Altogether, 62,397 of 951,428 reads were mapped to the mitochondrial genome, with the average mapped read length = 516.6 bp and average genome coverage = 5,061.5. Only the partial mapping result at the joint region is shown. Blank spaces between sequences denote borders of sequence reads, whereas colors of nucleotides indicate quality scores (dark nucleotides have higher quality score; minimum score = 30.0). The numbers above nucleotide sequences are positions in the mitochondrial genome (KP796149) from this study. The 3′ and 5′ ends of the mitochondrial genome of Cyclo_CDC_2013 (KP658101) are marked

Single contigs containing the complete mitochondrion genome were identified by Blast analysis of the assembly of sequences from the whole genome sequencing project. The overlapped ends of the contigs generated in different genome assembly efforts indicated that the mitochondrion genome was either circular or concatemeric. This was supported by read mapping at the joint region between two mitochondrial genome units (Fig. 6). Each mitochondrial genome unit is 6,229 bp in length with an AT content of 66.59 %. It codes the complete genes for cytb, cox1, and cox3, and fragments of the SSU rRNA and LSU rRNA, and has the same organization reported recently [20]. The full mitochondrial genomic sequence is 45 nucleotides longer than the published genome KP658101 (Fig. 6). Among the 45 nucleotides, 30 align with the 3′ terminus sequences from *Eimeria* spp. proposed by Ogedengbe and colleagues [20], with the conserved palindromic sequence ‘CTGTTATTTTGTC’ replaced by the sequence ‘CTGTATTTTTATTATTTAATTTTAC’. The remaining 15 nucleotides ‘TATTTTAAATAGTAT’ is upstream of the 21-bp A/T-only region in *C. cayetanensis*, which was suggested to be the beginning of the linear monomeric genome of mitochondria in *C. cayetanensis* [20]. They align better to the conserved 5′-terminus sequences of *Eimeria* spp. than the 21-bp A/T-only region. Thus, the published mitochondrial genome KP658101 has 15 and 30 nucleotides missing at the 5′ and 3′ ends, respectively.

#### Genetic similarity to other apicomplexans

Blast analysis indicated that *C. cayetanensis* has 87–92 % sequence similarities to the mitochondrial genomes of various *Eimeria* spp. in GenBank, with the highest similarity to the genome in *E. dispersa* (KJ608416). This was confirmed by direct comparison of the assembled *C. cayetanensis* and *E. tenella* genomes by using NUCmer, which showed an overall sequence identity of 90.4 %. Comparison of the mitochondrial genomes of apicomplexans indicated that the *C. cayetanensis* genome has complete synteny with genomes of *Eimeria* spp. (Fig. 3b). Analysis of SNV distribution by read mapping along the genome of *E. tenella* showed that the most conserved regions are nucleotide positions 2,800 to 4,900 and positions 5,400 to the end of the genome, where the rRNA gene fragments are located (Fig. 4b). Phylogenetic analysis of the mitochondrial genome sequences demonstrated that *C. cayetanensis* formed a clade with *E. dispersa* from turkeys and quails and *E. magna* from rabbits, sister to a clade formed by cecum-infecting *Eimeria* spp. from chickens and turkeys (Fig. 5b).

#### Sequence polymorphism in *C. cayetanensis* genomes

Eight single-nucleotide variants (SNVs) and one 7-bp multiple-nucleotide variant (MNV) were detected between the mitochondrial genomes of the *C. cayetanensis* isolate from this study (CHN_HEN01) and the isolate (Cyclo_CDC_2013 (KP658101) in the published study (Table 2). They are dispersed over the entire mitochondrial genome, including both coding and noncoding regions. Both cox1 and cox 3 genes have two SNVs. The 7-bp MNV near and within SSU9 is reverse-complemental in sequence between the two isolates (Table 2).

**Table 2:** Nucleotide sequence differences in the mitochondrial genome of *Cyclospora cayetanensis* between the Chinese isolate CHN_HEN01 (KP796149) and US isolate Cyclo_CDC_2013 (KP658101)

Nucleotide position^a^	Gene or region	Nucleotide in Cyclo_CDC_2013	Nucleotide in CHN_HEN01
60	SSU/8	T	A
2007	cox1	G	A
2253	cox1	G	A
3131	Intergenic between LSU/12 & SSU/1	T	A
3964	Intergenic between LSU/8 & SSU/5	C	A
4282	LSU/1	A	T
4703	cox3	C	T
4937	cox3	C	T
6085–6091	Intergenic between SSU/11 & SSU9 and within SSU9	TAATAAC	GTTATTA

^a^According to GenBank sequence KP658101

## Discussion

In this study, the complete apicoplast genome of *C. cayetanensis* has been sequenced for the first time. We have also obtained the last 45-bp sequence to complete the mitochondrial genome published recently [20]. The apicoplast and mitochondrial genomes of *C. cayetanensis* are both highly similar to those of *Eimeria* spp. in genome sizes and gene contents, with a complete synteny in genome organization. As seen in *Eimeria* spp. and other apicomplexans, the relatively large apicoplast genome of *C. cayetanensis* codes for the entire array of machinery needed for the expression of the apicoplast genome, including two copies of SSU rRNA and LSU rRNA, various ribosomal proteins, three RNA polymerases, and tRNAs for all 20 amino acids. Thus, *C. cayetanensis* should have a functional apicoplast organelle, which as in other apicomplexans likely plays key roles in the biosynthesis of fatty acids, isoprenoids, iron-suffer clusters and heme [12, 25]. Like in other coccidian parasites [26], the TGA codon for tryptophan is common in the apicoplast genome of *C. cayetanensis*. In contrast, hemosporidians such as *Plasmodium* spp., *Theileria* spp., *Babesia* spp., and *Leucocytozoo*n spp. do not have any in-frame TGA codons in their apicoplast genomes [25, 27]. One in-frame TGA codon was also seen in the mitochondrial cox3 gene in *C. cayetanensis.*

Phylogenetic analysis of both apicoplast and mitochondrial genomes from a diverse group of apicomplexans supports the close relationship between *C. cayetanensis* and avian *Eimeria* spp., thus confirming the results of direct comparison of organelle genome organization and those from previous phylogenetic analyses of the partial nucleic SSU rRNA and HSP70 genes [28–31]. However, some minor differences in results among phylogenetic studies have been observed. In the present study, phylogenetic analyses of the complete apicoplast and mitochondrial genomes both suggest that *C. cayetanensis* is more closely related to the cecum-infecting avian *Eimeria* spp. such as *E. tenella* and *E. necatrix* than to the small intestine-infecting avian *Eimeria* spp. such as *E. brunetti*. This is in agreement with the results of an analysis of the partial SSU RNA gene [30]. Other analyses of the SSU rRNA gene, however, have shown that *C. cayetanensis* formed a sister cluster with avian *Eimeria* spp. [29, 32]. Like in this study, most phylogenetic analyses of the SSU rRNA gene have shown a clear separation of the cecum-infecting avian *Eimeria* spp. from the small intestine-infecting *Eimeria* spp. [19, 29, 30, 32]. A recent analysis of the complete mitochondrial genomes of *Eimeria* spp. has also shown the formation of separate clusters by the cecum-infecting and small intestine-infecting avian *Eimeria* spp. [33]. Comparisons of the nucleic genomes of these parasites are needed to better understand the evolutionary position of *Cyclospora* spp. and the biologic significance of the relatedness between *C. cayetanensis* and cecum-infecting avian *Eimeria* spp.

The structure of the mitochondrial genome of *C. cayetanensis* in this study is different from the one proposed recently. Based largely on amplification failures using primers that have worked reliably for various *Eimeria* spp. and new primers designed based on the assumption of a circular or linear concatenated genome, Ogedengbe and colleagues have suggested recently that *C. cayetanensis* likely has a linear monomeric genome [20]. This is very differently from the linear concatenated genome of *E. tenella* identified based on restriction fragment length polymorphism analysis [34]. Results of our genome assembly and sequence mapping indicate that *C. cayetanensis* probably has the same mitochondrial genome structure as *E. tenella*. In the published study, the high sequence divergence between *Cyclospora* and *Eimeria* near the joint of two mitochondrial genome units was probably responsible for the amplification failures in the analysis of the sequence at the joint for *C. cayetanensis*.

Some sequence differences have been observed between mitochondrial genomes of this and the published studies. Altogether, there are eight SNVs and one MNV between genomes of the Chinese isolate examined in this study and the 2013 Texas outbreak isolate in the previous report [20]. Four of the SNVs are in the cox1 and cox3 genes. Previously, analysis of the full cox1 and cox3 genes showed no sequence differences among five *C. cayetanensis* isolates from limited areas (Southeast Asia and United States) [20]. Comparative analysis of the apicoplast and mitochondrial genomes has been used in tracking the spread of *P. falciparum* and *P. vivax* [16–18]. With further verifications, this approach can be potentially used in geographic tracking of *C. cayetanensis* during investigations of cyclosporiasis outbreaks in North America.

With the whole apicoplast and mitochondrial genome sequences available, better intervention measures and diagnostic tools can potentially be developed for *C. cayetanensis*. The close relatedness between avian *Eimeria* spp. and *Cyclospora* spp. indicates that avian *Eimeria* spp., especially those infecting the cecum, may serve as an alternative model for studying *Cyclospora* spp. Thus far, there are no feasible animal models for *C. cayetanensis* [35], and feeding sporulated *C. cayetanensis* oocysts to human volunteers did not produce any infection [36]. The genetic similarity between avian *Cyclospora* spp. and *Eimeria* spp. suggests that many of the drugs used in the treatment of poultry coccidiosis may be effective against *C. cayetanensis* infection. Other therapeutics specifically targeting the mitochondrial and apicoplast metabolism can be developed [13, 14, 37].

## Conclusions

In conclusion, data on the complete apicoplast and mitochondrial genomes of *C. cayetanensis* have been obtained for the first time. Both genomes are highly similar to those of cecum-infecting avian *Eimeria* spp., and sequence variations in the mitochondrial genome between two Chinese and US *C. cayetanensis* isolates have been identified. The availability of whole apicoplast and mitochondrial genome sequences would improve our understanding of the biology of *C. cayetanensis* and facilitate the development of new intervention tools. Further characterization of the genomes of other *Cyclospora* species and additional *C. cayetanensis* isolates is needed to improve our understanding of the taxonomic position of *Cyclospora* spp. and the development of genotyping tools for case linkage and infection/contamination source tracking in outbreak investigations.

### Ethics statement

The study was done under Human Subjects Protocol No. 990115 “Use of residual human specimens for the determination of frequency of genotypes or sub-types of pathogenic parasites,” which was reviewed and approved by the Institutional Review Board of the Centers for Disease Control and Prevention (CDC). No personal identifier was associated with the *C. cayetanensis* specimen at the time of submission for diagnostic service at CDC.
